# Prenatal exposure to the organophosphate insecticide chlorpyrifos enhances brain oxidative stress and prostaglandin E_2_ synthesis in a mouse model of idiopathic autism

**DOI:** 10.1186/s12974-016-0617-4

**Published:** 2016-06-14

**Authors:** Alessia De Felice, Anita Greco, Gemma Calamandrei, Luisa Minghetti

**Affiliations:** Section of Neurotoxicology and Neuroendocrinology, Department of Cell Biology and Neurosciences, Istituto Superiore di Sanità, Viale Regina Elena 299, I-00161 Rome, Italy; Section of Experimental Neurology, Department of Cell Biology and Neurosciences, Istituto Superiore di Sanità, Viale Regina Elena 299, I-00161 Rome, Italy; Present address: Center for Neuroscience and Cognitive Systems @UniTn, Istituto Italiano di Tecnologia, Via Bettini 31, 38068 Rovereto (TN), Italy

**Keywords:** Autism spectrum disorders, BTBR mice, Isoprostanes, Prostaglandins, Lipid metabolism, Oxidative stress, Neuroinflammation, Pesticides

## Abstract

**Background:**

Autism spectrum disorders (ASD) are emerging as polygenic and multifactorial disorders in which complex interactions between defective genes and early exposure to environmental stressors impact on the correct neurodevelopment and brain processes. Organophosphate insecticides, among which chlorpyrifos (CPF), are widely diffused environmental toxicants associated with neurobehavioral deficits and increased risk of ASD occurrence in children. Oxidative stress and dysregulated immune responses are implicated in both organophosphate neurodevelopmental effects and ASD etiopathogenesis. BTBR T+tf/J mice, a well-studied model of idiopathic autism, show several behavioral and immunological alterations found in ASD children, and we recently showed that CPF gestational exposure strengthened some of these autistic-like traits. In the present study, we aimed at investigating whether the behavioral effects of gestational CPF administration are associated with brain increased oxidative stress and altered lipid mediator profile.

**Methods:**

Brain levels of F_2_-isoprostanes (15-F_2t_-IsoP), as index of in vivo oxidative stress, and prostaglandin E_2_ (PGE_2_), a major arachidonic acid metabolite released by immune cells and by specific glutamatergic neuron populations mainly in cortex and hippocampus, were assessed by specific enzyme-immuno assays in brain homogenates from BTBR T+tf/J and C57Bl6/J mice, exposed during gestation to either vehicle or CPF. Measures were performed in mice of both sexes, at different postnatal stages (PNDs 1, 21, and 70).

**Results:**

At birth, BTBR T+tf/J mice exhibited higher baseline 15-F_2t_-IsoP levels as compared to C57Bl6/J mice, suggestive of greater oxidative stress processes. Gestational treatment with CPF-enhanced 15-F_2t_-IsoP and PGE_2_ levels in strain- and age-dependent manner, with 15-F_2t_-IsoP increased in BTBR T+tf/J mice at PNDs 1 and 21, and PGE_2_ elevated in BTBR T+tf/J mice at PNDs 21 and 70. At PND 21, CPF effects were sex-dependent being the increase of the two metabolites mainly associated with male mice. CPF treatment also induced a reduction of somatic growth, which reached statistical significance at PND 21.

**Conclusions:**

These findings indicate that the autistic-like BTBR T+tf/J strain is highly vulnerable to environmental stressors during gestational period. The results further support the hypothesis that oxidative stress might be the link between environmental neurotoxicants such as CPF and ASD. The increased levels of oxidative stress during early postnatal life could result in delayed and long-lasting alterations in specific pathways relevant to ASD, of which PGE_2_ signaling represents an important one.

## Background

The etiological bases of the majority of human neurodevelopmental disorders, including autism spectrum disorders (ASD), are still unknown though a great body of data supports their polygenic and multifactorial etiology [1]. Substantial evidence indicates that ASD may derive from the complex interaction between many defective genes and early exposure to different environmental stressors, which can alter the typical neurodevelopment with consequent disruption of some behaviors [2].

Dysregulated immune responses and lipid metabolism impairment, associated with enhanced oxidative stress, are thought to contribute to etiopathogenesis of ASD and other neurodevelopmental disorders [3–5]. In particular, some data suggest that alterations in lipid metabolism, due to multiple causes, play an important role in autism pathogenesis [6, 7]. Oxidative stress, a condition defined as an alteration in the balance between pro-oxidant and anti-oxidant molecules and associated with long-term damage [8], is one of the plausible mechanisms that link genes and environment. Several data point to a key role of oxidative stress in human toxicity of industrial chemicals, such as heavy metals and pesticides [9–11]; in particular, induction of oxidative stress, besides acetylcholinesterase (AChE) inhibition, is among the main mechanisms mediating both acute and chronic organophosphate (OP) pesticide exposure [9, 10, 12]. The human brain is composed predominantly by lipids; polyunsaturated fatty acids (PUFAs) such as arachidonic acid, eicosapentaenoic acid, and docosahexaenoic acid are major constituents of the cell membranes. PUFAs can be metabolized through different enzymatic pathways to generate lipid signaling messengers [13]. Among them, prostaglandin E_2_ (PGE_2_), an arachidonic acid metabolite of the cyclooxygenase (COX) pathway, is an important lipid signaling messenger affecting many immune and brain functions [14].

PUFAs are exquisitely sensitive to oxidative stress, and broad range metabolites can be generated non-enzymatically, by direct free radical attack of membrane esterified PUFAs [15]. Among these lipid peroxidation products, F_2_-isoprostanes, and in particular 15-F_2t_-Isoprostane (15-F_2t_-IsoP, also known as 8-isoprostane-F_2α_), are considered reliable index of in vivo oxidative stress. Of note, increased levels of 15-F_2t_-IsoP have been detected in both red blood cells and urine samples of children with autism compared to age-matched controls [16]; later, El-Ansary and co-workers found elevated plasma F_2_-isoprostanes in Saudi autistic children, together with alterations in lipid mediators such as PGE_2_ and leukotrienes [17].

Black and tan brachyury T+tf/J (BTBR) is an inbred mouse strain that displays several behavioral traits relevant to autism, such as impairments in social and communication domains with lacks of sociability in social approach tasks, reduction in the emission of ultrasonic vocalizations in various social settings, and high levels of repetitive behaviors [18]. Although well-studied, the inherited genetic changes that lead to autistic-like behaviors in these mice are scarcely known and still under active investigation [19–21]. BTBR mice show several of the immunological alterations found in children with ASD, such as an increased rate of immunological activation, with high levels of pro-inflammatory cytokines such as IL-1β, IL-6, and TNF-α and increased number of microglia at the adult stage [22, 23].

The link between immune system dysregulation and behavioral deficits in ADS is not yet well understood, and the BTBR mouse provides a well-established model to further investigate this issue. Immune disturbances found in this mouse strain might result in higher vulnerability to oxidative stress promoted by toxicants, with possible more severe repercussions on neural development, a hypothesis that has also called in to explain the environmental contribution to autism risk in children [24].

To test this hypothesis, in the present study, we administered the OP pesticide chlorpyrifos (CPF) at the sub-toxic dose of 6 mg/kg/body weight or vehicle to pregnant mice of either C57 BL6/J (C57) or BTBR inbred strains, by oral gavage, from gestational day (GD) 14 to 17, replicating as for dose and administration schedule our previous studies performed in CD-1 outbred mice [25, 26]. Among OP pesticides, CPF has recently been listed as one of the environmental chemicals possibly responsible for increased ASD risk in children [27]. Specifically, epidemiological studies indicate that environmentally relevant exposure in utero to CPF may affect children’s neuropsychological maturation, in terms of impaired reflex functioning in newborns [28, 29] and decreased mental and psychomotor performances and attention problems in infants, with higher risk to develop pervasive developmental disorders [30]. In agreement with human data, an increasing body of rodent data indicates that, at sub-toxic doses, developmental exposure to CPF affects neurobehavioral maturation, targeting neural and neuroendocrine systems involved in ASD etiology [31–35]. In the present study, C57 mice were used as control strain since they present strong similarity with BTBR mice as for gene background, but lack the autism-like phenotype [19]. 15-F_2t_-IsoP and PGE_2_ were measured in the whole brain at postnatal day (PND) 1 and 70 in the offspring of both sexes in the two strains. The same parameters were measured at weaning (PND 21) in the BTBR strain. BTBR mice showed higher baseline levels of 15-F_2t_-IsoP at birth as compared to C57 mice, suggestive of enhanced oxidative stress processes. In line with our hypothesis, gestational CPF treatment selectively enhanced oxidative stress in BTBR mice at birth (PND 1) and weaning (PND 21), whereas PGE_2_ levels were particularly elevated at adulthood (PND 70).

## Methods

### Animals

Male and female mice of the BTBR and C57 strain purchased from the Jackson Laboratory (Bar Harbour, ME, USA) were housed upon arrival in breeding cages (polycarbonate cages 33x13x14 cm) under standard animal housing conditions (temperature 20 ± 2 °C; humidity 60–70 %) with food (enriched standard diet for mice from Altromin, Spezialfutter GmbH & Co. Germany) and water ad libitum, under a 12:12 reverse light cycle (lights on from 8:00 p.m. till 8:00 a.m.). Females were inspected daily for the presence of the vaginal plug (GD 0). On GD 14, pregnant females were randomly assigned to one of the two prenatal treatments (vehicle, CPF). CPF (Chem. Service, West Chester, PA) was dissolved in peanut oil as vehicle to provide rapid and complete absorption. CPF (in a volume of 0.1 ml/10 g at a dose of 6 mg/kg/bw) or its vehicle was administered to pregnant females from GD 14 to 17 by intraoral gavage. The dose of CPF was originally selected on the basis of previous multidose studies performed in our laboratory in CD1 mice [26, 36, 37] as the more effective one in producing behavioral changes in the absence of overt toxic symptoms in dams or major effects on pregnancy length, number of pups at delivery, sex ratio, pups’ weight at delivery and growing rate. This same dose was able to induce significant changes in early motor development and amplification of the autism-like behavioral traits in adult males in BTBR strain (see below). A total of 31 litters (16 vehicle-treated and 15 CPF-treated) were used. Females’ body weight was monitored daily during pregnancy. Proportion of term pregnancies, gestation length, litter size, sex ratio, and neonatal mortality were also measured to exclude potential effects of the treatment on reproductive performances. On the day of birth, which is defined as PND 0, the sex of the pups was assessed by evaluation of anogenital distance. One pup of each sex was randomly selected from each litter and sacrificed at either PND 1, PND 21 (BTBR only), or PND 70 for the assessment of 15-F_2t_-IsoP and PGE_2_ in the brain. The siblings of the BTBR mice used in the present study formed the subjects of a different study aimed at describing early and delayed effects of CPF on the behavioral phenotype of BTBR mice [25]. At weaning (PND 21), male and female offspring were separated and maintained in same-sex pairs till the adulthood. Body weight of each individual pup was recorded on PNDs 1, 4, 6, 8, and 12, at weaning (PND 21) and at adulthood (PND 70). In addition, nine C57 mice and ten BTBR mice born from mothers not receiving any treatment were used to measure the baseline levels of both 15-F_2t_-IsoP and PGE_2_ in the two strains on the day of birth.

### 15-F_2t_-IsoP and PGE_2_ measurement

The levels of 15-F_2t_-IsoP were assessed as previously described [38]. Briefly, brains were weighed and homogenized in 50-mM Tris buffer, pH 7.5 (1 mg/0.1 ml), containing the anti-oxidant BHT (10 μM) and the COX inhibitor indomethacin (1 μM) to block ex vivo arachidonic acid auto-oxidation and PGs formation. Homogenates were vigorously vortexed and incubated for 5 min on ice before centrifuging at 14,000 rpm for 45 min at 4 °C. Supernatants were collected and stored at −80 °C until assayed. 15-F_2t_-IsoP was measured by a specific competitive enzyme immunoassay (Cayman Chemical Company, Ann Arbor, MI), according to the manufacturer’s instructions. Ellman’s reagent was used as a substrate and absorbance measured at 405 nm using a microplate reader (GDV DV990BV6). For quantification, a standard curve was built with eight serial dilutions ranging from 500 to 0.8 pg/ml and analysis performed using four-parameter logistic fitting. Detection limit was 2 pg/ml; anti-15-F_2t_-IsoP antibody cross-reactivity with other iso-prostaglandins was less than 0.15 %. PGE_2_ levels were measured by a competitive high sensitivity enzyme immunoassay (sensitivity 8.25 pg/ml; Assay Design, Inc., Ann Arbor, MI). A buffered solution of p-nitrophenyl phosphate was used as substrate and absorbance measured at 405 nm with correction at 570 nm, using a microplate reader (GDV DV990BV6). For quantification, a standard curve was built with eight serial dilution ranging from 1000 to 7.8 pg/ml and analysis performed using four-parameter logistic fitting. Anti-PGE_2_ antibody cross-reactivity with other prostaglandins was less than 1.5 %. Serial dilutions of samples were then tested, in duplicate, for 15-F_2t_-IsoP and PGE_2_ content.

### Statistical analysis

Data are expressed as means ± SEM of *n* independent experiments (run in duplicate). Due to the large difference in baseline levels between birth and adulthood, each age point was analyzed separately. Statistical significance was evaluated applying factorial ANOVA with strain (2 levels), treatment (2 levels), and sex (2 levels) as fixed grouping factors. Multiple comparisons were performed by the Tukey HSD test. Correlation coefficients (*r*_s_) were calculated by Spearman’s rank correlation. *p* < 0.05 was accepted as statistical significance. Data were analyzed using the Stata Tm 10 statistical package (Stata Corporation, College Station, TX, USA).

## Results

### Brain levels of 15-F_2t_-IsoP and PGE_2_ in BTBR and C57BL6/J newborn mice

**Fig. 1:**
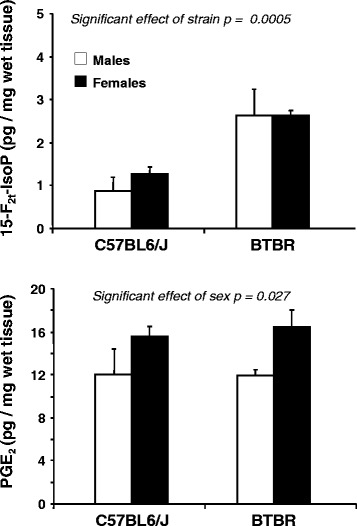
Brain levels of 15-F_2t_-IsoP and PGE_2_ in untreated male and female C57BL6/J and BTBR mice at PND 1. Levels of 15-F_2t_-IsoP or PGE_2_ in brain homogenates are given as pg/mg of wet tissue and are expressed as mean ± SEM (*n* = 4 males and 5 = females for both BTBR and C57BL6/J)

Firstly, we compared brain levels of both 15-F_2t_-IsoP and PGE_2_ at PND 1 in BTBR and C57 mice of both sexes born to untreated mothers (Fig. 1). BTBR mice showed higher 15-F_2t_-IsoP levels than C57 mice [main effect of strain: *F*(1,14) = 20.607, *p* = 0.0005], suggesting that, in line with the observations reported in ASD children, BTBR mice are characterized by higher levels of oxidative stress. PGE_2_ levels were not significantly different between the two strains of mice, but they were higher in female offspring of both strains [main effect of sex: *F*(1,14) = 6.029, *p* = 0.027].

### Effects of CPF prenatal exposure in BTBR and C57BL6/J mice

In agreement with our previous studies, the selected dose for CPF treatment (6 mg/kg/bw) did not affect reproductive parameters such as gestation length, weight gain of the pregnant females, and weight and viability of the pups at birth, in both strains [25].

**Fig. 2:**
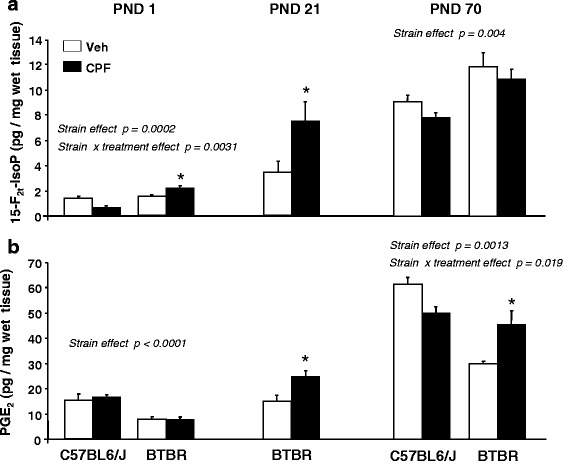
Effects of CPF prenatal exposure on 15-F_2t_-IsoP and PGE_2_ brain levels in C57BL6/J and BTBR mice at PNDs 1, 21, and 70. Brain levels of 15-F_2t_-IsoP (**a**) and PGE_2_ (**b**) were measured in brain homogenates of C57BL6/J (PND 1 and 70) and BTBR mice (PND 1, 21, and 70) prenatally exposed to CPF or vehicle from GD 14 to 17. Data are given as pg/mg of wet tissues and are expressed as mean ± SEM; *p < 0.05 significant difference between vehicle and CPF treatment (*n* = 10–12 for BTBR and *n* = 6 for C57BL6/J).

The effect of gestational CPF administration on brain levels of 15-F_2t_-IsoP and PGE_2_ is shown in Fig. 2. As for 15-F_2t_-IsoP (Fig. 2a), at PND 1, ANOVA yielded a significant effect of strain [*F*(1,31) = 17.519, *p* = 0.0002] with BTBR mice showing higher levels of 15-F_2t_-Isop than C57 mice and a significant two-way interaction between strain and treatment [*F*(1,31) = 10.323, *p* = 0.0031]. BTBR mice prenatally exposed to CPF exhibited increased levels of 15-F_2t_-IsoP as compared to vehicle-exposed BTBR mice (*p* < 0.05 after post hoc comparisons), while CPF treatment tended to decrease 15-F_2t_-IsoP brain levels in C57 strain (this difference just missed statistical significance). Brain levels of PGE_2_ were significantly higher in C57 than in BTBR mice (Fig. 2b) [*F*(1,31) = 36.75, *p* < 0.0001] and were not influenced by CPF treatment in either strain. In BTBR pups born to vehicle-treated mothers, the levels of both 15-F_2t_-IsoP and PGE_2_ were decreased as compared to the offspring of untreated mothers (see Fig. 1), an effect that could be related to reported higher susceptibility to handling stress of BTBR than C57 mice [39]; the effect was, however, transient, and no differences were appreciated between offspring of naive and vehicle-treated mothers at later time points (not shown).

At PND 70, we found a significant main effect of the strain [*F*(1,26) = 9.947, *p* = 0.004] as BTBR mice had higher levels of 15-F_2t_-IsoP than C57 mice, regardless the treatment received during gestation (Fig. 2a, right panel). PGE_2_ levels (Fig. 2b) were still higher in C57 than in BTBR strain [main effect of strain *F*(1,24) = 13.18, *p* = 0.0013], but ANOVA yielded a significant interaction between strain and treatment [*F*(1,24) = 6.329, *p* = 0.019], as CPF induced a significant increase of PGE_2_ in the BTBR strain (*p* < 0.05 after post hoc comparisons) and a decrease, which did not reach significance, in the C57 strain.

At PND1 and PND 70, there were no sex differences for either 15-F_2t_-IsoP or PGE_2_ levels in both CPF- and vehicle-exposed (BTBR or C57 groups, not shown).

### Effects of CPF prenatal exposure in BTBR mice at PND 21

**Fig. 3:**
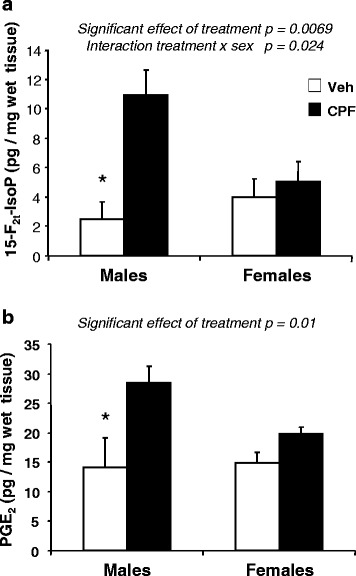
Effects of CPF prenatal exposure in male and female BTBR mice at PND 21. Brain levels of 15-F_2t_-IsoP (**a**) and PGE_2_ (**b**) were measured in brain homogenates of male and female BTBR mice prenatally exposed to CPF or vehicle. Data are given as pg/mg of wet tissues and are expressed as mean ± SEM; #*p* < 0.05 vs vehicle-exposed mice (*n* = 6–8 per group)

On the basis of above findings showing different profiles of effects at the neonatal stage and adulthood in BTBR mice only, we sought to further investigate the effect of gestational CPF treatment in this strain at PND 21. This age corresponds in laboratory mice to the adolescent phase, namely to a critical transition point in which the second wave of neuronal reorganization occurs, through mechanisms of pruning to eliminate the synapses in excess [40, 41]. We found that at this age, CPF treatment significantly increased the levels of both 15-F_2t_-IsoP and PGE_2_ (Fig. 2). For both 15-F_2t_-IsoP and PGE_2_, ANOVA yielded a significant main effect of treatment at this age [15-F_2t_-IsoP: *F*(1,10) = 11.468, *p* = 0.0069; PGE_2_: *F*(1,10) = 12.309, *p* = 0.0056]. Furthermore, in the case of 15-F_2t_-IsoP (Fig. 3a), a significant interaction between treatment and sex [*F*(1,10) = 7.025, *p* < 0.024] evidenced that the effect of CPF was limited to the male sex (*p* < 0.05 after post hoc comparisons). Since a similar trend was observed also for PGE_2_, we decided to perform post hoc comparisons on the interaction between sex and treatment [*F*(1,9) = 2.486, *p* = 0.14], as the Tukey HSD test can be used also in the absence of significant ANOVA results [42]. We found that the effects of CPF were limited to the male sex also in the case of PGE_2_ (*p* < 0.05 after comparison between vehicle and CPF within the male group).

### Effects of CPF prenatal exposure on somatic growth in BTBR and C57BL6/J mice

In a previous study, we monitored the effect of CPF prenatal exposure on somatic growth of BTBR mice between PND 4 and 12, showing a negative trend and significant effect at PND 12 [25]. As shown in Table 1, body and brain weight of both C57 and BTBR mice prenatally exposed to CPF was not significantly different from that of vehicle-exposed mice at PND 1 and PND 70. At PND 21, body weight of BTBR mice prenatally exposed to CPF was significantly lower than that of vehicle-exposed mice at this age [*F*(1,12) = 10.99, *p* = 0.0062], while no significant effects were observed on brain weight (Fig. 3b, right). When considering all three ages, 15-F_2t_-IsoP and PGE_2_ brain levels and brain weights in BTBR mice were positively correlated in both vehicle (*r*_s_ 0.57, *p* = 0.0011 and *r*_s_ 0.60, *p* = 0.0003, respectively)- and CPF-exposed mice (*r*_s_ 0.76, *p* = 0.0000; *r*_s_ 0.81, *p* = 0.0001, respectively). Similarly, in C57 mice, 15-F_2t_-IsoP and PGE_2_ brain levels and brain weights were positively correlated in both vehicle (*r*_s_ 0.67, *p* = 0.017 and *r*_s_ 0.68, *p* = 0.0153, respectively)- and CPF-exposed mice (*r*_s_ 0.84, *p* = 0.003; *r*_s_ 0.7, *p* = 0.007, respectively).

**Table 1:** Somatic and brain growth in C57BL6/J and BTBR mice prenatally exposed to CPF

		C57BL6/J	BTBR	C57BL6/J	BTBR
		Body weight (g)		Brain weight (mg)	
PND 1	Vehicle	1.75 ± 0.052	1.61 ± 0.084	75 ± 5	66 ± 3
	CPF	1.68 ± 0.097	1.67 ± 0.120	70 ± 9	63 ± 2
PND 21	Vehicle	nt	14.30 ± 0.88	nt	157 ± 6
	CPF	nt	11.07 ± 1.05*	nt	149 ± 6
PND 70	Vehicle	23.92 ± 1.76	30.71 ± 1.62	297 ± 18	327 ± 12
	CPF	24.68 ± 1.69	29.4 ± 1.33	307 ± 8	317 ± 12

Data are means ± SEM (*n* = 10–12 for BTBR and *n* = 6–7 for C57BL6/J); *p < 0.05 versus vehicle-exposed BTBR mice at same PND

*nt* not tested

Somatic and brain growth were similar in both sexes (not shown).

## Discussion

Raising evidence points to oxidative stress as a major pathogenic mechanism shared by etiological determinants of neurodevelopment disorders such as genetic and environmental factors. Several data confirm that induction of oxidative stress is a major contributor of the overall toxicity of the OP pesticides [43]. In particular, acute exposure of adult rodents to high doses of CPF has been reported to increase lipid peroxidation and to decrease the levels of key enzymes implicated in oxidative defenses [44–47]. However, recent epidemiological evidence raised concern on the impact that the prolonged exposure to apparently non-toxic doses of OP in critical phases of brain maturation might have on child’s neurodevelopment. Experimental models mimicking the human exposure scenarios to this widely diffused class of pesticides may be pivotal to elucidate the mechanisms implicated in OP developmental neurotoxicity and identify novel biomarkers for risk assessment.

To the best of our knowledge, this is the first study analyzing the effect of gestational exposure to a sub-toxic dose of CPF on brain levels of 15-F_2t_-IsoP, a reliable and sensitive marker of in vivo oxidative stress, in mice, at birth and later stages corresponding to adolescence and adulthood*.* In addition to 15-F_2t_-IsoP, we evaluated the levels of PGE_2_, a lipid mediator involved in inflammatory and immune responses but also controlling several physiological and pathological functions in the brain and found altered in ASD.

Our findings indicate that at birth, BTBR mice, a validated model of idiopathic autism displaying both behavioral and immunological deficits associated with ASD, are characterized by increased brain levels of 15-F_2t_-IsoP as compared to C57 mice, a strain with strong gene background similarity but lacking of autism-like phenotype. Gestational exposure to CPF promoted the increase of both 15-F_2t_-IsoP and PGE_2_ in BTBR mice but not in C57 mice, suggesting an important strain-treatment interaction effect and a selective vulnerability of the BTBR strain to this organophosphate.

Interestingly, the increase in 15-F_2t_-IsoP and PGE_2_ levels occurred with different temporal patterns, being 15-F_2t_-IsoP elevated at birth and at weaning and PGE_2_ levels not affected at birth but stably increased at weaning and adulthood. This suggests that the occurrence of oxidative stress in a specific temporal window in early postnatal life might result in delayed permanent alterations in specific molecular pathways relevant to ASD, such as PGE_2_ signaling [6, 7, 17].

PGE_2_ is a major arachidonic acid metabolite, preferentially formed during the enzymatic activity of COX-2 [48], the inducible COX isoform. Besides its well-known role in inflammation, COX-2/PGE_2_ pathway plays important physiological functions. In normal brain, COX-2 is localized to excitatory glutamatergic neurons and its expression is dependent on synaptic activity [49]. COX-2 expression is developmental regulated and in the neocortex COX-2 positive neurons show a typical laminar distribution that is heavily disrupted in subjects affected by Rett syndrome, a neurodevelopmental disorder characterized by a defective development of cortical neurons [50]. It is tempting to speculate that the increased PGE_2_ levels after prenatal CPF exposure could reflect an exacerbation of the functional abnormalities evidenced in young/adult BTBR mice, which include widespread reductions in cortical thickness and reduced fronto-cortical metabolism [51].

In the only study measuring F_2_-IsoPs and PGE_2_ in rats acutely exposed to CPF, the former were positively associated with learning deficits induced by CPF [47].

Though the design of our present study does not allow a direct correlation between the altered brain levels of 15-F_2t_-IsoP and PGE_2_ and autism-like behavioral, it is worth noting that in the siblings of the experimental subjects used in the current study, CPF gestational exposure caused a significant delay in motor maturation in the first 2 weeks of life and worsened some of the autistic-like traits of BTBR mice at adulthood [25]. Anomalies in specific motor responses, common to other mouse models of ASD, have been proposed as early markers for ASD diagnosis [52–54] strengthening the relevance of our findings [25].

In light of the strong prevalence of ASD and other neurodevelopmental disorders in the male sex, it is worth considering that the effects of CPF are of comparable extent in the two sexes both at birth and at adulthood. However, on PND 21, there is a significant sex effect, with males displaying the highest levels of 15-F_2t_-IsoP and PGE_2_. This observation supports a higher vulnerability of males to prenatal exposure to CPF and is consistent with sex differences in CPF behavioral effects, suggested by epidemiological and experimental studies. Horton et al. [55] found that male children were more susceptible than females as for cognitive effects, and we reported higher vulnerability of the male sex to developmental CPF effects, particularly as far as early neonatal behavior patterns is concerned [25].

A higher vulnerability of the male sex to adverse conditions associated with oxidative stress is indicated by a study in newborn twins, a human natural model for studying fetal adaptation to suboptimal supply of nutrients—the most likely cause of reduced fetal growth—in which occurrence of oxidative stress has been demonstrated. In this study, males displayed higher cord blood levels of 15-F_2t_-IsoP than females; the observation held true when comparing twins of unlike-sex pairs, strongly suggesting that males are more exposed to oxidative stress than their sisters when experiencing the same “in utero” environmental challenge [56].

Strong evidence indicates that males are more sensitive to early-life challenges, such as infection and ischemia, and it has been suggested that this may contribute to sexual dimorphism evident in early-onset developmental disorders, including autism ([57] and references therein).

In line with our previous results [25], CPF-exposed BTBR mice showed a reduced somatic growth in the developmental phase. Both CPF-induced oxidative stress and reduced body weight reached the apex at weaning, to return to normal level at adulthood, suggesting that reduced growth rate and increased brain oxidative stress might be part of the same adaptive response to adverse conditions during early phases of life. Although the effect on somatic growth was similar in males and females, the increase in brain oxidative stress was higher in males, further supporting the particular vulnerability of the male sex to developmental suboptimal conditions. The reduction in body weight resulting from CPF treatment at PND 21 in BTBR mice was not associated with a corresponding reduction in brain weight, an observation that might suggest a transient increase in brain size with respect to body weight during the developmental phase. Interestingly, a transient brain overgrowth has been reported in some studies on ASD children ([58] and references therein), in which overgrowth and neural dysfunctions have been suggested to underlie some of the autistic symptoms.

Altogether, we show here that a widely diffused insecticide reportedly implicated in increased risk of neuromotor and neuropsychological impairments in children induces a transient up-regulation of an oxidative stress biomarker and a permanent alteration in PGE_2_ pathway in a validated mouse model of ASD. A possible limitation of our study is the use of a single CPF dose. Importantly, the CPF dose used in the current and previous studies is safe with respect to reproductive performance of treated dams (pregnancy length, number of pups at delivery, sex ratio); it does not induce systemic toxicity in dams or major effects on pup’s health, and it is below the threshold for inhibition of brain AChE in the mouse species [26, 59]. Our findings thus reinforce the growing evidence that the developmental neurotoxicity of CPF is not strictly or uniquely related to its anticholinesterase activity [60]. However, there is an indication of potential non-monotonic dose-response effects of gestational CPF on behavioral functions [61]. Even within the range of sub-toxic doses, in critical maturational phase, “higher” doses may cause small and transient enhancement of cholinergic neurotransmission: such event might be paradoxically protective for the developing brain insofar that compensatory mechanisms are called upon. Thus, we cannot exclude that a lower dose of CPF would have evidenced a different pattern of effects in the two strains considered.

## Conclusions

In the last years, numerous studies have suggested that immunological factors as well as increased susceptibility to oxidative stress may contribute to the etiology of ASD.

Our findings indicate that prenatal CPF exposure, at a dose devoid of maternal or systemic toxicity, significantly affects oxidative stress and PGE_2_ synthesis in a validated mouse model of idiopathic autism. In the light of the hypothesis associating oxidative stress, lipid metabolism alterations, and ASD etiology in children, future work in this mouse model of autism will help in elucidating the mechanistic pathways linking exposure to a widely diffused neurotoxicant such as CPF, neurodevelopment alterations, and behavioral deficits.

## Abbreviations

15-F_2t_-IsoP, 15-F_2t_-Isoprostane; AChE, acetylcholinesterase; ASD, autism spectrum disorders; BTBR, black and tan brachyury T+tf/J; C57, C57BL6/J; CPF, chlorpyrifos; COX, cyclooxygenase; GD, gestational day; OP, organophosphate; PG, prostaglandin; PGE_2_, prostaglandin E_2_; PND, postnatal day; PUFAs, polyunsaturated fatty acids.
